# Endometrial cancer - reduce to the minimum. A new paradigm for adjuvant treatments?

**DOI:** 10.1186/1748-717X-6-164

**Published:** 2011-11-25

**Authors:** Heike R Scheithauer, Diana S Schulz, Claus Belka

**Affiliations:** 1 Universitiy of Munich - LMU, Department of Radiation Oncology, Marchioninistrasse 15, 81377 Munich, Germany

## Abstract

**Background:**

Up to now, the role of adjuvant radiation therapy and the extent of lymph node dissection for early stage endometrial cancer are controversial. In order to clarify the current position of the given adjuvant treatment options, a systematic review was performed.

**Materials and methods:**

Both, Pubmed and ISI Web of Knowledge database were searched using the following keywords and MESH headings: "Endometrial cancer", "Endometrial Neoplasms", "Endometrial Neoplasms/radiotherapy", "External beam radiation therapy", "Brachytherapy" and adequate combinations.

**Conclusion:**

Recent data from randomized trials indicate that external beam radiation therapy - particularly in combination with extended lymph node dissection - or radical lymph node dissection increases toxicity without any improvement of overall survival rates. Thus, reduced surgical aggressiveness and limitation of radiotherapy to vaginal-vault-brachytherapy only is sufficient for most cases of early stage endometrial cancer.

## Introduction

Over years there has been a constant debate regarding the extent of the lymph node dissection and the role of adjuvant treatments for localized endometrial carcinoma. Especially the role of adjuvant radiation treatment has not yet been well defined.

An early analysis from the GOG-database documented a clear correlation between tumour size, tumour grade and the subsequent risk of local failure, loco-regional failure and distant seeding [1,2]. Thus complex pattern of parallel and competing risks exist.

In order to reduce the vaginal vault failures and lymph node failures brachytherapy alone, percutaneous pelvic radiation alone or in combination with afterloading procedures has been employed [3-8].

Until now the final impact of these strategies on overall survival has only been weakly defined.

In parallel, it has been speculated that local control may be increased by an extended lymph node dissection [9-12]. Again, the precise value of increased surgical aggressiveness has not been validated.

Aim of the article is to critically review the available data and the recently published randomized trials.

### Major trials and randomized radiotherapy trials

At present the data of the following prospective or randomized trials determining the role of radiation as adjunct to surgical procedures for patients with endometrial carcinoma are available:

Poulsen and co-workers published the oldest trial regarding the use of radiation therapy in 1997 [13,14]. The Danish group analysed the outcomes in patients prospectively chosen to receive no external radiation in pT1 low risk (n = 641) whereas pT1 high risk (n = 235), stage II (n = 105) and stage III group 1 (n = 58) were treated regularly.

Tumour recurrences occurred in 7% in stage I low-risk patients, 15% in stage I high-risk patients, 29% of stage II, and 47% of stage III patients. The conclusion was to omit external radiotherapy from treatment concept for patients with low risk endometrial cancer.

However, a major shortcoming of the trial was the missing of randomization. Thus several randomized trials were initiated.

The Gynaecological Oncology Group (GOG-99 trial) was designed to analyse the value of adjuvant percutaneous radiotherapy in patients with stage I and stage II endometrial carcinoma. The surgical procedure was stated to have included lymphadenectomy (LAE). However, the extent of LAE (number of node retrieved, lymph node areas dissected) was not documented.

Although the trial was initially designed as prospective trial, it became apparent that the targeted patient population was at a lower risk for recurrence than initially expected. Therefore, the initial definition of risk groups was replaced. Thus, on a formal basis the trial cannot be considered as pure prospective trial.

Using the ex post risk definition, patients with high-risk features had an increased rate of recurrences which translated into a reduced overall survival rate. High risk was defined as a complex combination of age and other risk factors (moderately to poorly differentiated tumours, lymph vessel invasion, outer third invasion). High-risk phenotype was defined as age > 70 and one other factor, > 50 with any two of the other risk factors, or any age with three of the other risks. Patients with these constellations benefited significantly from pelvic irradiation [7,15].

In parallel, the PORTEC study group also aimed to determine the impact of pelvic irradiation without additional brachytherapy on the outcome in patients with early stage endometrial cancer [8].

In this regard the surgical procedure was a simple total abdominal hysterectomy and bilateral salpingo-oophorectomy (TAH-BSO) without any lymphadenectomy. Patients were mainly diagnosed with pT1b G2 31%, pT1b G3 10%, pT1c G1 20%, pT1c G2 39%, pT1c G3 < 1%.

Without randomisation, patients with a pT1c G3 carcinoma were treated routinely with pelvic irradiation and were followed in a register-only arm of the trial [16].

In principle, pelvic irradiation was found to be important for local control (HR 3,9; 2.0-7,6), however, omission of radiotherapy did not impact on overall survival (0,76; 0,4-1,4).

When analysing this trial in detail it becomes obvious that at least one third of the patients had only a very limited risk for pelvic lymph node involvement. Thus, it is not surprising that the increase in local control does not frankly translate into a survival benefit.

Patients with pT1c G3 (n = 99) routinely received pelvic irradiation resulting in 5% vaginal relapse and pelvic relapses in 7%. The fate of these patients was dominated by a high distant failure rate (31%). Thus, even in absence of an extended surgical procedure local control is adequate [16].

Long term follow up of this trial confirmed the initial interpretation that pelvic radiotherapy is an overtreatment for low to intermediate risk patients [17]. This is of special importance when bearing in mind that pelvic irradiation is associated with a mildly but clearly increased risk of side effects [18]. Taken together, although the PORTEC-1 trial was designed to answer the question which cohort would benefit from pelvic irradiation, it finally failed to really define a clear subset of patients in need of pelvic irradiation. Several other questions were not addressed by the trial: Since in parallel with the upcoming of the randomized data, several cohorts of patients treated with brachytherapy only were reported, the evident question was in how far local vaginal radiotherapy alone would be suitable [19-21].

Not directly related to the use of radiotherapy both randomized trials left open the question in how far the degree of lymphadenectomy would influence the use of radiotherapy and finally, the question in how far distant failure would be decreased by adding aggressive poly-chemotherapy.

The PORTEC-2 and the MRC ASTEC and NCIC CTG EN.5 trial represent the logical continuation of the aforementioned research issues. Whereas the PORTEC-2 trial analysed the role of pelvic irradiation compared to vaginal vault brachytherapy, the ASTEC/EN.5 group initiated a complex trial aiming to define the role of both, adjuvant pelvic radiotherapy and systematic pelvic lymphadenectomy.

#### ASTEC/EN.5 radiotherapy trial

For a better understanding of radiotherapy and of lymph node dissection the NCIC and the MRC designed a double randomization based trial setting (referred to as ASTEC, "A Study in the Treatment of Endometrial Cancer" for the MRC part of the trial and "EN.5" for the NCIC part of the trial) [22,23].

In a first approach, patients with endometrial cancer were randomized to receive TAH-BSO with or without lymphadenectomy. In a subsequent step patients with intermediate or high-risk early cancer were randomly assigned to pelvic irradiation versus no pelvic irradiation. Vaginal vault brachytherapy was done based on the individual centre policy. Main endpoint of the trial was overall survival.

The majority of patients were diagnosed pT1c (24% G1, 37% G2, 11% G3). The collective included sufficient numbers of patients harbouring a reasonable risk of lymph node seeding. Pelvic irradiation significantly reduced the number of local and loco-regional relapses. However, in absolute numbers, the level of risk reduction was low (29/453 versus 13/452).

The major result of the trial showed that overall survival with external beam radiotherapy was not better than after observation (hazard ratio 1.05; 95% CI 0.75-1.48; p = 0.77), both groups had a 5-year overall survival rate of 84%. In the framework of this trial the authors performed a meta-analysis of all previous trials confirming that there is no benefit in terms of overall survival (hazard ratio 1.04; 95% CI 0.84-1.29). Statistically an absolute benefit of external beam radiotherapy at 5 years of more than 3% could be readily excluded.

However it has to be taken into account that roughly 50% of the patients in the observation arm received vaginal vault brachytherapy leading to an underestimation of the net effects of irradiation.

Since the rate of distant failures was merely equal in both arms the small improvements in local control did not translate into an improvement of overall survival. In addition, the trial revealed a mildly but clearly visible increase in early and late toxicity.

Nevertheless, some criticism was issued especially in regard to patient selection biases [24]:

Creasman and co-workers commented, "Only one third of the surgical patients from the first study were actually secondarily randomized to radiation therapy". Furthermore "Patients could have lymph node metastasis confirmed on pathology yet be randomized to no external beam radiation therapy (EBRT)".

In addition, only less than one third of the patients had lymph node dissection and of those less than 20% had an adequate dissection. Thus the majority of the patients may have been not adequately staged. A further bias is generated by the fact that brachytherapy was used in roughly 50% of the patients assigned to the observation arm. Since brachytherapy is efficiently reducing the local relapse rate putative effects of pelvic irradiation may be diluted.

#### PORTEC-2 radiotherapy trial

The initial PORTEC-1 trial documented that pelvic irradiation in the absence of a systematic lymphadenectomy increased local control in low-intermediated risk patients, however overall survival was not influenced and toxicity was increased [17]. Based on these results, the Dutch consortium commenced a new randomized trial addressing the question in how far whole pelvic irradiation or brachytherapy alone is sufficient for patients with intermediate risk endometrial carcinoma [25,26].

The study collective (n = 427) comprised patients with pT1b, pT1c carcinomas grade 1 and grade 2. Surgery was TAH-BSO without any additional lymph node dissection. Primary endpoint was vaginal vault failure with overall survival and quality of life being secondary endpoint. 5-year rates of vaginal recurrence were calculated as followed: Vaginal brachytherapy 1.8% (95% CI 0.6-5.9) vs. 1.6% (95% CI 0.5-4.9) after pelvic radiotherapy (HR EBRT 0.78, 95% CI 0.17-3.49; p = 0.74). The combination of vaginal and pelvic failures revealed 5-year rates of loco-regional recurrence of 5.1% (2.8-9.6) after brachytherapy, and 2.1% (0.8-5.8) for EBRT (HR 2.08, 0.71-6.09; p = 0.17). Rates of distant metastases were similar in both arms. Key observation was the fact that no differences in overall or disease-free survival occurred. However, acute grade 1-2 gastrointestinal toxicity was significantly lower in brachytherapy when compared to the EBRT patients. A subsequent analysis of health-related quality of life (HRQOL) issues and side effects confirmed these results [27].

Similar to the ASTEC/EN.5 trial several issues are still not solved by this trial. After central pathology review it turned out that most of the patients suffered from deeply invasive grade 1 cancer. Thus the translation of these results to the general collective of intermediate risk patients may not be justified [28].

In her comment P. Eifel concluded that "even with more than 400 patients, PORTEC-2 was probably statistically underpowered; in part, because of the large proportion of relatively low-risk, grade 1 tumours, the total number of events was small" [28].

#### Other major recent analysis

Since all given randomized trials do not completely answer the open questions in how far pelvic radiotherapy improves overall survival after endometrial carcinoma a SEER data base analysis was undertaken: In this regard, 56.360 eligible patients were identified with 70.4% low, 26.2% intermediate, and 3.4% high risk. A proportion of 41.6% underwent LAE and 17.6% adjuvant RT. In low-risk disease, RT was not associated with increased survival whereas in intermediate-risk disease lymphadenectomy and RT (80.6% RT vs. 74.9% no RT, p < 0.001) were associated with higher survival without differences between RT modalities. In high-risk disease lymphadenectomy and irradiation were associated with increased survival. In the absence of lymphadenectomy pelvic irradiation was superior to brachytherapy only (p = 0.01)[29].

#### Role of lymphadenectomy

The value of systematic lymphadenectomy for adequate staging is well accepted for many cancer sites. In contrast, final prove is lacking that an extended lymphadenectomy is able to impact on overall survival.

In case of endometrial carcinoma, several non-randomized trials including a SEER data based survey indicated that the number of removed lymph nodes correlates with an increased overall survival [9,12].

These finding were the rationale for increasing the aggressiveness of the surgical approach in several countries. However, any analysis based on SEER data may harbour a massive bias. In this regard, the SEER analysis revealed that the number of lymph nodes removed also correlated to a reduction of the non-tumour related death rates [12,30]. Thus it seems likely that surgeons tended to remove more nodes in healthier patients.

In order to avoid the inherent shortcomings from retrospective approaches and SEER data base analysis, randomized trails had to be performed. In this regard the results from two major trials have been published recently [23,30].

The Italian trial randomized 514 patients with preoperative stage I endometrial carcinoma to undergo pelvic systematic lymphadenectomy (n = 264) or no lymphadenectomy (n = 250). The primary endpoint of the trial was overall survival with disease-free survival and surgical morbidity being secondary endpoint. No influence on overall survival was detectable. However, surgical staging was improved with more positive lymph nodes detected. In parallel, the toxicity was significantly increased. The use of radiation and other adjuvant treatments were at the discretion of the treating physician. In the lymphadenectomy arm 44 patients (16.7%) received irradiation whereas in the observation arm 63 patients (25.2%) were treated with radiotherapy.

In a similar approach, the ASTEC/EN.5 trial randomized 1408 women with histologically proven endometrial carcinoma confined to the corpus to surgery (hysterectomy and BSO; n = 704) or surgery plus lymphadenectomy (n = 704). Again, the primary endpoint of the trial was overall survival. The reported absolute difference in 5-year overall survival was 1% (95% CI -4 to 6). After adjustment for baseline characteristics and pathology details, no significant differences in either overall survival or recurrence free survival were detectable. Data on acute or late toxicity were not reported.

The issue in how far the interpretation of the ASTEC trial may be blurred by the fact that a relevant part of the patients received radiotherapy (~ 25%) may be answered by taking the results of the PORTEC-2 trial into account: The PORTEC-2 study collective was similar to the cohort treated inside the ASTEC trial with one major difference: Lymph-node sampling was not performed at all - nevertheless outcomes in term of local control DFS and OS were similar. Thus, the results from PORTEC-2 strongly support the interpretation from the ASTEC lymph-node dissection trial.

A frequently discussed limitation of both trials is the fact that a systematic removal of the para-aortal nodes has not been performed. Since the initial analysis by Creasman and Alders [1,31] it is known that, in parallel to the increasing risk for pelvic lymph node metastasis, the proportion of positive para-aortic lymph node increases. A recent retrospective Japanese trial analysed outcomes (overall survival) in 671 patients with endometrial carcinoma treated with either pelvic lymphadenectomy (n = 325 patients) or pelvic and para-aortic lymphadenectomy (n = 346). Additional adjuvant treatments were offered to patients with intermediate or high risk of recurrence [32].

Overall survival was better in those patients with pelvic and para-aortic lymphadenectomy when compared to pelvic lymphadenectomy alone (HR 0.53, 95% CI 0.38-0.76; p = 0.0005). No difference was detectable between intermediate or high-risk patients whereas overall survival was not influenced by lymphadenectomy type in low-risk patients. In multivariate analysis pelvic and para-aortic lymphadenectomy reduced the risk of death compared with pelvic lymphadenectomy in intermediate to high-risk patients (HR 0.44, 0.30-0.64; p < 0.0001).

## Conclusion and future developments

Although the recent data add substantial new aspects for guiding the treatment of patients with endometrial cancer, many aspects are still ill-defined. The randomized data on either radiotherapy or systematic lymphadenectomy indicate that the general impact of a more aggressive approach on overall survival in most patients is rather limited. However, there are enough hints pointing to the interpretation that in certain subgroups a more aggressive approach is advisable. Unfortunately - at present - these groups are not defined. It is likely that patients with high-risk cancer need an optimal local treatment in parallel to effective systemic treatment. The outcome of patients suffering from pT1c G3 treated within the observational arm of the PORTEC-1 trial indicates that surgery and radiation finally lead to high local control rates however many of those patient fail distantly. Vice versa, strategy with surgery plus chemotherapy may be associated with unacceptably high local failure rates [33].

Merely for all low-risk patients the data point to a more minimalistic approach. Taken into account that all PORTEC data were based on a simple TAH-BSO without any lymphadenectomy and none of the prospective trials documented any benefit of extended surgery, surgery may be limited to a TAH-BSO. Adjuvant radiotherapy may also be limited to vaginal vault irradiation.

Still all prospective trials are subject to certain criticisms: It is frequently stated that the validity of the Italian trial as well as the ASTEC trial is limited by the fact that irradiation was used in a substantial number of the Italian patients and the absolute number of nodes removed was not perfectly high. However, at least for the cohort included in the trial the results of the PORTEC-2 trial make systematic removal of nodes questionable. In addition the general value of the PORTEC-2 is partially questioned based on the high proportion of patients with grade 1 cancer.

The value of either the ASTEC EN.5 trial, the Italian trial and also the Japanese cohort study is limited by complex biases introduced by the uncontrolled use of additional adjuvant treatment or crossovers.

Thus at present the treating clinician should be aware of the data suggesting a reduced aggressiveness for low-risk patients. In parallel the crucial recommendation issued by P. Eifel in her recent position paper should be followed strictly: "The PORTEC-2 trial underscores the critical role of pathologists and other specialists in the management of uterine carcinomas. These cancers are best managed in an environment where there is an experienced multidisciplinary team of gynaecologic oncologists, radiation oncologists, and gynaecologic pathologists. Management by less experienced clinicians can result not only in confusing trial results but also in inappropriate therapies, treatment delays, and added costs to society".

## Competing interests

The authors declare that they have no competing interests.

## Authors' contributions

HS, DS and CB performed the database search, critically reviewed the existing data and drafted the manuscript. All authors have read and approved the final manuscript.
